# Cognitive rationalization in occupational fraud: structure exploration and scale development

**DOI:** 10.3389/fpsyg.2023.1112127

**Published:** 2023-07-06

**Authors:** Miao Yang, Yizao Chen

**Affiliations:** ^1^School of Accounting, Liaoning University of International Business and Economics, Dalian, China; ^2^School of Accounting, Shandong University of Finance and Economics, Jinan, China

**Keywords:** cognitive rationalization, occupational fraud rationalization, structure exploration, scale development, criterion validity

## Abstract

The structure and measurement of occupational fraud rationalization as one of the motivations for fraudulent behavior has been a major obstacle in theoretical research and practical problems. In order to answer the fundamental question, “What does cognitive rationalization of occupational fraud involve?,” this paper explored the structure and scale development of the internal psychological factors of occupational fraud rationalization. Several research methods were used for this purpose, such as data collection, research interviews, review & verification, project purification, structural verification, and reliability & validity test. The results showed that, based on the internal structure, occupational fraud rationalization presented second-order three-dimensional and first-order eight-dimensional factors. Further, a formal scale containing 27 items related to the structure of the metric was constructed to measure the occupational fraud rationalization. In terms of variable correlations, this paper empirically tested the criterion validity of occupational fraud rationalization from the perspective of personality traits. The result revealed a significant positive (negative) correlation between Machiavellian traits (empathy traits) and occupational fraud rationalization, respectively. In conclusion, this paper provides an attempt to address the cognitive and measurement challenges of occupational fraud rationalization, expanding the application and development of moral disengagement theory in the field of occupational fraud and laying some groundwork for subsequent research and development.

## 1. Introduction

The term “rationalization” was first coined by Welsh neurologist Jones in 1908, who defined rationalization as the logic and reasoning behind rational individual behavior choices. In early research, psychologists generally agreed that rationalization represented a psychological process whereby rational behavior was achieved through faulty motivation (Sloane, 1944). This psychological process only occurred when the actor could not reasonably restrain their behavior, and rationalization could provide a plausible excuse for such deviant behavior that arose. In real practice, rationalization originated in the development of criminology, which was applied as an explanation and tool in the field of occupational fraud. Cressey’s (1953) study of white-collar professional crime found that rationalization was present in almost every perpetrator’s explanation of his criminal behavior. This phenomenon was summarized in the neutralization theory proposed by Sykes and Matza (1957), where the term “neutralization” suggested that rationalization was a psychological mechanism used to hedge and mitigate internal moral condemnation. On this basis, Bandura (1986) interpreted rationalization as moral disengagement from a social psychological perspective. However, in essence, both neutralization theory and moral disengagement are the same manifestation of rationalization in different fields of study, and thus their essence is consistent (Murphy and Dacin, 2011). This indicates that occupational fraud rationalization is an application concept that applies rationalization to the field of occupational fraud. Chen et al. (2019) defined occupational fraud rationalization as the cognitive process by which individuals give self-persuasive reasons to deviate their behavior from their ethical perceptions in order to reduce the negative effect of occupational fraud on their sense of ethics.

The problem of occupational fraud has always been an incurable “tumor” in the field of corporate governance with its high incidence, high hazard and high concealment. It seriously impacts the operation of the national economy and exacerbate the inequitable distribution of social wealth. As such, it has also undoubtedly become a constraint to high-quality economic development, national governance capacity and modernization of the governance system. For this reason, both academic and practical circles have devote themselves to seeking the causes of occupational fraud and good ways to govern it. Arguably since the emergence of modern corporate governance structures, the most discuss and practice theory on the causes of occupational fraud is the fraud triangle theory proposed by Cressey (1953). This theory states that pressure, opportunity and cognitive rationalization together constitute the three core factors in the governance of occupational fraud. In the process of decision fraud, the motivation for fraud is driven by the pressure factors. From the perspective of fraud prevention, the control of the opportunity factor focuses on hard constraints such as external supervision and the building of internal control systems, which fall under the category of exogenous governance. In contrast, the cognitive rationalization factor focuses on the moral self-discipline of the actor, which is essentially in the realm of internal governance. Cognitive rationalization explains the psychological process of “why an actor commits an immoral act” from a moral psychological perspective.

However, due to the fact that cognitive rationalization of occupational fraud is a potentially psychological dimensional variable, it is difficult to capture, measure and control. Further, this has led to the challenge in conducting in-depth research on this factor of fraud governance at this stage. Throughout domestic and international research, the internal structure of occupational fraud cognitive rationalization has not been explored in a more systematic way, leaving a gap in the basic question of “what does occupational fraud cognitive rationalization consist of.”

At present, there is no systematic understanding of the perceived reasonableness of the perpetrator in committing fraud in either the theoretical or practical community. The explanations of cognitive rationalization of professional fraud are only given in the form of examples, as seen in the US Statement on Auditing Standards No. 99 (Consideration of fraud in a financial statement audit) and China Auditing Standards for Certified Public Accountants No. 1141 (Fraud-related liabilities in the audit of financial statements). Even in the fraud triangle theory, the presentation of cognitive rationalization is relatively superficial and does not provide sufficient depth of theoretical knowledge. Therefore, systematically revealing the “inner structure and quantification methods of occupational fraud cognitive rationalization” is undoubtedly a prerequisite for promoting the development of research on cognitive rationalization factors at both the theoretical and practical levels. Therefore, this paper aims to explore and validate the internal structure of occupational fraud cognitive rationalization through a systematic and standardized testing procedure. Based on this, a reliability-tested occupational fraud cognitive rationalization scale will be developed in an attempt to break the current “one-sided and fragmented” status quo in this area of research.

## 2. Theoretical basis and literature review

### 2.1. Measurement and assessment of the occupational fraud rationalization

#### 2.1.1. Self-reporting method

The self-reporting method shows the real psychological activity of the fraud perpetrator by interacting whit the perpetrator at the time of committing the fraud by interacting their behavior. In Murphy’s (2012) and Mayhew and Murphy’s (2014) experiments, the system automatically pops up the “Why did you misrepresent your income” dialog box to capture the true thoughts of the research subjects when they commit fraud. The self-reporting method captures the psychological thoughts of the research subject quickly and honestly with high reliability. However, its disadvantages are also evident. Firstly, the measurement process is cumbersome. The method must be repeated multiple times to achieve reasonable human classifications. Secondly, the unexpected dialogues can cause stressful emotions such as revulsion and disgust in fraud perpetrator, which can easily bias the experimental results. In addition, a significant limitation is that the results obtained by this method are less reproducible and generalizable. The self-report method is therefore a suitable research tool for the early exploratory phase of research.

#### 2.1.2. Scale measurement method

##### 2.1.2.1. Balanced inventory of desirable responses (Paulhus deception scales)

Desai et al. (2010) chose to use the balanced inventory of desirable responses (BIDR) to measure the rationalization tendencies of fraudsters. The scale was developed by Paulhus (1984) as a tool to measure the social approval effects. The social approval is a behavior that seeks social approval in the form of social expectations by highlighting positive features of the self by denying or concealing true thoughts (or behaviors) of the self that are not appreciated. However, cognitive rationalization is a mechanism used to alleviate the internal self-condemnation and therefore, in terms of essential connotations, the two do not coincide.

##### 2.1.2.2. Neutralization scale based on the neutralization theory

Initially, this scale covered only five common rationalization mechanisms (Sykes and Matza, 1957). Due to its limited coverage, it was further researched and refined by subsequent researchers in different fields using this scale (Ball, 1973; Garrett et al., 1989). Slezak (2013) used the neutralization scale from Ball’s (1973) version to measure the rationalization tendency for occupational fraud. The Ball neutralization scale contains nine methodological dimensions. While such complementary empirical findings have contributed to the refinement of the rationalization mechanisms, to date there is no single research dimension for rationalization research based on neutralization theory. The number of rationalization dimensions in different studies depends on the literature they cite, which leads to biased conclusions and difficulties in cross-sectional comparisons across studies (Chen et al., 2019).

##### 2.1.2.3. Moral disengagement scale based on the moral disengagement theory

The scale was originally created for children or adolescents and has since been used as a basis for researchers in different fields to develop or revise scales appropriate for specific situations, such as the military (Mcalister et al., 2006), sport (Boardley and Kavussanu, 2008), and the organizations (Barsky, 2011). The most significant advantage of the moral disengagement scale over the neutralization scale is that it presents a more stable, intrinsic mechanism comprising eight items (Bandura, 1999). Unfortunately, however, on the one hand, there is no scale for the field of occupational fraud to date.; on the other hand, due to their unique historical background and cultural heritage, Chinese people exhibit distinctive ethical and cultural values in the process of work transactions than in Western countries (Pan et al., 2014), Therefore, simply copying Western theory for a scale that is hoped to be used in Chinese research is tantamount to “cutting the foot to fit the shoes.”

#### 2.1.3. Antecedent substitution method

The antecedent substitution method stimulates an individual’s specific rationalization cognition by using and controlling for situations or behaviors that induce particular type of rationalization (Brown, 2014). This is a situational research method that lends itself to a single cognitive rationalization and can greatly enhance the understanding of causal relationships between variables. However, it has a disadvantage is that it only allows for the study of a single dimension of specific cognitive rationalization tendencies can be studied. Therefore, it is not suitable for a comprehensive picture of cognitive rationalization factors within the context of occupation fraud.

In summary, there are a variety of methods for measuring the rationalization of occupational fraud, and the choice of method varies between researchers, illustrating the phenomenon of “one person chooses one method.” Each method, of course, has its own advantages and disadvantages. Specifically, the self-reporting method is an exploratory method that may not be suitable for widespread use. The antecedent substitution method is only applicable to single scenario studies and it is difficult to tailor to the requirements of various studies. Among the scale measures, the BIDR has serious internal misalignment, while the neutralization scale has no uniform methodological dimension. As for moral disengagement theory, there is no scale based on this theory specifically designed for occupational fraud scenario, nor does it take into account the ethical culture and values specific to China. It can therefore be concluded that no research has yet explored and validated the internal structure of the cognitive rationalization of occupational fraud in a more systematic way, so this paper attempts to undertake preliminary work in this area.

## 3. Initial construction of occupational fraud rationalization scale

### 3.1. Data collection and investigation

To fully demonstrate the specific concrete manifestations of occupational fraud rationalization and to conceptualize it as a quantifiable item, this paper began with a comprehensive information collection and survey interviews. These include, (1) existing scales—this refers to a review and systematic collation of the current literature on ethical disengagement scales. (2) We summarized and integrated the valuable literature from both domestic and international sources. With references to Tsang (2002), Murphy and Dacin (2011), Murphy (2012), Moore et al. (2012), Mayhew and Murphy (2014), Chen et al. (2017), Reinstein and Taylor (2017) and Chen et al. (2019). (3) Information was collected in internet search engines using “occupational fraud,” “occupational corruption,” “occupational crime,” and “occupational misappropriation” as keywords. Reference was also made to the announcement of administrative penalties given by the China Securities Regulatory Commission (CSRC) and the information published on the China Judgment Online. (4) Reference was made to books such as “21st Century major listed companies at home and abroad fraud Case Study: Why would a first-class enterprise and talent fraud? “, “Analysis of Listed Companies’ Fraud: Based on the Perspective of Fraud Triangle Theory,” “Anti-Corruption Warning Book for Senior Officials,” “Anti-Corruption Warning Book—60 Examples of Integrity Reminders,” “Anti-Corruption Warning Book 2—Psychological Analysis of Corruption” etc. (5) Fraud perpetrator interviews. After filtering, interviews were conducted with 21 incarcerated fraud officers from the Economic Crime Investigation Section of a municipal Public Security Bureau. It is worth stating that, to present a complete picture of the specific representations of the occupational fraud perception rationalization, the 21 interviewees selected for this study had a relatively even distribution of high and low positions. Basic information on the cases involving these 21 individuals is shown in Table 1.

**Table 1:** Occupational fraud interview case statistics.

Case type	Number of cases	Average loss value (10 thousand)
Passive bribery of non-state agents	5	17
Loan fraud	2	765
Fraudulent invoicing	3	739
Illegal loan	9	184
Misstatement of registered capital	2	790

The categories of occupational fraud cases covered in this interview include, passive bribery of non-state agents (staff of a company, enterprise or other unit using the convenience of their positions to solicit or illegally accept property from others), loan fraud (obtaining loans from banks or other financial institutions by means of falsified information, etc.), fraudulent invoicing (using the convenience of one’s position to obtain illegal benefits by issuing false invoices for others when the facts of purchase and sale do not exist), Illegal loan (financial institutions issuing loans in violation of state or corporate regulations), and misstatement of registered capital s (individuals or units using false documents or other fraudulent means to register companies).

In line with the requirements of theoretical research method, interviews should not set uniform assumptions and patterns in advance. However, a simple outline can be outlined to improve the quality and efficiency of the interview (Pettigrew, 2000). The average length of this interview was between 30 to 45 min. Prior to the interview, each interviewee was informed about the recorded interviews and privacy-related issues, and was assured that no information would be disclosed. During the formal interview, we asked them to elaborate on their specific fraud process in order to recall the true psychological motivations and emotional feelings at the scene of the crime. We also asked questions such as, “What drove you to do this?,” “Did you feel uneasy while doing it?,” to understand the person’s motivation for the fraud and to capture the cognitive rationalization process. For the explanations given by the person, it was determined that explanations based on pressure (e.g., I want to gain the benefit) and opportunity (e.g., I have the opportunity to commit fraud) were not considered as cognitive rationalizations, based on Murphy’s (2012) three-element framework of fraud triangle theory. The whole interview process was kept interactive and open. After the interview, according some explanations for cognitive rationalization were collated and extracted, as follows:

“I did it to help the company to tide over its difficulties and to keep employees from losing their jobs, not for myself.”

“In our industry, things like this (taking bribes and gifts) are very normal human affairs.”

“Those business owners are outwardly justified, but behind the scenes they are actually corrupt (bribery)?”

“Do you think I want to do that? What can I do if I do not do it when my boss is pointing a gun at me?”

“I did it (misrepresented the registered capital) so that the company could grow quickly.”

### 3.2. Structural exploration and topics collation based on grounded research

Based on the theoretical approach of the qualitative study, this paper collated and coded the cognitive rationalization scenarios for the five types of occupational fraud mentioned above. The sequence of “open coding → axis coding → selective coding” was followed. Firstly, the different scenarios were organized to extract concepts. Secondly, similar images are linked and clustered through induction and deduction. Finally, through integration and condensation, the attachment points of the core categories of each category are found to form a complete explanatory framework.

Eight first-order conceptual categories covering 102 items were initially developed through organization and coding, as follows:

#### 3.2.1. Moral justification

Moral attributes are assigned to deviant behavior by reinterpreting it as a higher social value or social ethic, e.g., “I falsified the financial statements for the sake of the company.”

#### 3.2.2. Euphemistic label

Fraud is described as usual or harmless behavior through carefully chosen rhetoric, e.g., “Accepting gifts is an exchange of favors in the workplace.”

#### 3.2.3. Favorable comparison

Demonstrating the acceptability of one’s fraudulent behavior by comparing it with more egregious behavior, e.g., “Accepting a small favor is nothing compared to huge amounts of corruption.”

#### 3.2.4. Transfer responsibility

Giving the psychological implication that “the fault is not mine” by shifting responsibility for the fraud to the person giving the instructions or issuing the orders, e.g., “It was a superior’s decision to whitewash the company’s financial statements and I, as the forced enforcer, should be exempt.”

#### 3.2.5. Dilute responsibility

Weakening individual moral responsibility by shifting responsibility for fraud to the group or environment, e.g., “It makes no sense to focus on my individual actions when the whole organizational environment is full of corruption.”

#### 3.2.6. Emphasis on results

Excusing oneself with the idea that one’s fraudulent actions did not hurt anyone or had only a negligible impact, e.g., “I got some benefit but no one suffered any loss.”

#### 3.2.7. Differentiation

Dividing the group around oneself into one’s own people (internal people) and non-self (external people) and rationalizing one’s fraudulent behavior by demeaning the victim as an external person, e.g., “I had nothing to do with him, he was the one who was incompetent.”

#### 3.2.8. Guilty victim

The psychological burden of committing fraud is alleviated by citing the victim’s fault, e.g., “My hard work was exploited by the company, so by committing fraud, it’s just a matter of getting some compensation, which is no big deal.”

On this basis, further sorting revealed that the above eight first-order conceptual categories can be distilled into higher-order conceptual levels: (1) moral justification, euphemistic labeling and favorable comparison focus on the “nature of the fraud itself.” Specifically, the cognitive reinterpretation of behavior to make occupational fraud appear acceptable. The three categories above are thus grouped into the second-order conceptual category “cognitive reconstruction.” (2) The focus of transfer responsibility, dilute responsibility and emphasis on results is “assumption of liability for fraud.” Therefore, they are grouped into the second-order conceptual category “responsibility distortion.” (3) Both differentiation and guilty victim focus on the “victim of fraud,” by degrading the character or worth of the victim, the fraud is made to appear insignificant or deserved. The two above are therefore merged into the second-order conceptual category of “value devaluation.”

In summary, on the basis of induction and deduction, this paper achieved an initial structural exploration of the rationalization of the perception of occupational fraud. It contains three second-order dimensions: cognitive reconstruction, responsibility distortion and value devaluation. Among them, the first-order constructs of the cognitive reconstruction dimension include moral justification, euphemistic labeling and favorable comparison. The first-order constructs of the responsibility distortion dimension include transfer responsibility, dilute responsibility and emphasis on results. The first-order constructs of the value devaluation dimension include differentiation and guilty victim.

#### 3.2.9. Content self-inspection and review checks

According to Xi’s (2016) criteria for judging the quality of the scale, this paper proposes to conduct content self-check and review checks of the 102 items initially developed, and the inspection criteria of specific item content are shown in Table 2.

**Table 2:** Project content inspection standards.

Inspection Criteria	Specific requirements
Conciseness of items	Avoid lengthy items, but not at the expense of content
Clarity of expression	Avoid vague pronouns and inappropriate modifiers
Uniqueness of the item	Items that do not express two or more ideas at the same time
Readability of items	Readability level is appropriate at grade 5–7 level

To begin with, each of the 102 items initially formed was read and screened, and those that contained too many ideas, were vaguely expressed and poorly comprehensible were eliminated or revised. In the end, 64 items were obtained. To further examine the items, the reverse categorization method was used for the review. First, the 64 question items above were disrupted to form a reverse-categorization checklist, and three accounting PhD. students who were not involved in this research were invited to perform back-to-back independent categorization. The dimensional classification was explained to them before the test began to ensure that they understood the meaning accurately, after which the test takers read each item and judged the category to which it belonged. The detailed results are shown in Table 3. Of these, for the number of items classified in the expected category, 39 (61.%) were for three people, 12 (19%) were for two people, 9 (14%) were for one person and 5 (8%) were not classified in the expected category by anyone. It can be assumed that items with a high degree of inconsistency may be due to the fact that they contain multiple factors and are difficult to judge. Therefore, the 14 items with a result type of one person correctly classified with zero people were excluded. For those items that were correctly classified by the two, experts in the field of occupational fraud research were invited to make a final determination, and nine items were retained. This, together with the 38 items correctly classified by three people, resulted in a final total of 47 items.

**Table 3:** Results of review and verification of reverse categorization.

Number of people correctly categorizing items	Number of items	Percentage (%)
3	38	59
2	12	19
1	9	14
0	5	8

To ensure the readability of the items, three students (one in grade 6 and two in grade 7) were randomly invited to take a readability test of the items. The test was conducted independently. Testers were asked to read the items one by one and to comment on the comprehensibility of the items. The results were as follows: the grade 6 student said that he did not understand the term “fraud,” but otherwise had no comprehension problems. One grade 7 student stated that he had to read an item multiple times to understand it, while another grade 7 student said that he did not have any comprehension problems. The results suggested that the readability level of the r items was in the appropriate range.

After the above series of procedures, this paper initially constructed a 47-item occupational fraud rationalization scale. The paper then collected primary data by distributing questionnaires and used the quantitative data to achieve purification and structural validation of the scale items.

## 4. Pre-investigation and purification of scale items

A preliminary survey was conducted to ensure the quality of the scale was future refined through a preliminary survey. Questionnaires were distributed online to employees of the company through *www.wjx.cn*, and 226 valid questionnaires were returned. The items were scored using a seven-point Likert scale. The seven-point Liker scale is the most commonly used type of rating summation scale, it consists of a set of statements includes seven answer for one question. Every question has seven different answers strongly agree, agree, comparative agree, uncertain, comparative disagree, disagree, very disagree, and it records score 7 to 1 separately. The total score for each respondent’s attitude is the sum of answers to each question, it can indicate the strength of respondent’s attitude on this scale. Since occupational fraud rationalization is a sensitive research issue, respondents might be affected by social desirability effects. Fraud is a sensitive research question, although this questionnaire has been anonymity, but it is likely to be affected by the social approval effects. Even if the respondent’s answered in the direction of social expectations, masking their own unappreciated true thoughts of not being praised. To overcome this problem, this paper introduced Yang’s (1996) social desirability scale. The scale has 10 items, such as “Sometimes I cannot control myself and get angry with others,” with answers of “never” being scored 0 and “ever” being scored 1. Using Chen’s et al (2017) criteria, samples with a total score of social desirability effect less than 5 are more likely to fail to answer the questions according to their true intention, so they were excluded. On this basis, samples with too short a filling time were excluded, resulting in a final purified sample size of 175. The standard of too short is that time from beginning of answer to completion of the submission which lees than 2 minutes.

### 4.1. Item identification purification

In order to ensure that each item was clearly differentiated, it was necessary to conduct a differentiation analysis (Weng et al., 2018). This was done by adding up the scores of all items, sorting them from highest to lowest and dividing the top 27.% and bottom 27% of items into high and low scoring items, respectively. The scores of the items were tested for differences and if there was a significant difference between the groups (*p* < 0.05), the item was retained, otherwise, the item was excluded. In this paper, the 47 questions included in the scale were all significantly different.

The standard deviation of items reflects the volatility of scores within a single item. If the standard deviation of an item is too low, it indicates low volatility of the item score, i.e., poorer item discrimination (Liden and Maslyn, 1998). Therefore, items with a standard deviation of less than 0.50 should be excluded according to theoretical requirements (Weng et al., 2018). The results showed that the minimum standard deviation of 1.245 was met for all 47 items of the scale. Based on the above analysis, the item of the initial scale questionnaire were well differentiated.

### 4.2. Project reliability purification

To ensure that the overall scale has high reliability, the initial scale items were subjected to reliability purification through reliability analysis in this paper. The Cronbach’s *α* coefficient was used for the overall test index of the scale. Nunnally (1978) suggested 0.70 as the minimum acceptable value for the *α* coefficient. Xi (2016) synthesized the published scales and suggested the significance of alpha coefficients as follows: below 0.60, unacceptable, 0.60 to 0.65, not good enough, 0.65 to 0.70, minimum acceptable level, 0.70 to 0.80, good, 0.80 to 0.90, excellent, more than 0.9, excellent but the scale length can be considered to be shortened. The results showed that the overall reliability of the initial scale was 0.969, which was much more significant than 0.9, indicating that the overall reliability of the scale was excellent and that a shorter scale length could be considered.

In addition, the corrected total item correlation coefficient (CITC coefficient) was used to examine the level of reliability of individual items. In general, items with a CITC coefficient of less than 0.50 should be removed (Churchill, 1979). After several tests until all items had a CITC coefficient greater than 0.50, a total of 5 items were removed, the Cronbach’s *α* coefficient increased to 0.97, and the initial scale items were reduced from 47 to 42.

### 4.3. Factor analysis purification

To ensure the purity of the factor analysis, the scale items met the structural quality requirements on the remaining 42 items. KMO and Bartlett test results show that the sample data were suitable for factor analysis (KMO = 0.94, Sig. = 0.00). The research used principal component analysis (PCA) to extract common factors and obtained the factor loading matrices by the maximum variance method. According to the project checking standards (Pan et al., 2014), the following items were excluded sin order, (1) item commonality less than 0.50, (2) item factor loadings less than 0.50; (3) item cross-factor loadings greater than 0.40. The data analysis results were as follows, 0 items were removed because the item commonality was less than 0.50.4 items were removed because the item factor loadings were less than 0.50.6 items were removed because the project cross-factor loading were more than 0.40, leaving a final total of 32 items.

Finally, this paper returned to some of the test subjects. Subjects were consulted on their feelings about the item design and, based on the feedback, the presentation of three items was modified to improve the overall reading fluency. In the next stage, this article will conduct a formal survey through the refined occupational fraud rationalization scale, and plans to conduct a structural verification using the collected sample data to test the reliability and validity.

## 5. Structural validation and reliability testing of the perceived rationalization of occupational fraud rationalization scale

### 5.1. Data collection and sample distribution

The formal research was targeted at employees of the company and was distributed online via *www.wjx.cn*. The questionnaire were completed anonymously and the preamble of the questionnaire stated that the information obtained would be used for academic research only and would remain absolutely confidential. A total of 806 questionnaires were returned and the final valid sample size was 612 according to the same exclusion criteria as the pre-study. The demographic distribution of the sample was as follows. In terms of gender, 41.7% were male and 58.3% were female. In terms of age, 14.2% were aged 18 to 25, 34.8% were aged 25 to 30, while 33.5% were aged 30 to 40, 11.1% were aged 40 to 50, and 6.2% were aged 50 and above. In terms of educational, 10.6% were junior college and below, 32.7% were undergraduates and 56.7% were postgraduates. In terms of working years, 66.1% were 10 years and below, 20.8% were 10–20 years, and 13.1% were 20 years and above. In terms of position level, 70.1% were ordinary employees and 29.9% were managers and senior managers. In terms of annual income, 52.4% were below ¥100,000, 27.5% were between ¥100,000 and $200,000, 8.5% were between ¥200,000 and $300,000, and 7.4% were above ¥300,000. In terms of religious beliefs, 91.5% were non-religious and 8.5% had religious beliefs. In terms of the nature of the shareholding of the companies to which they belonged, 60.6% were non-state-owned enterprises and 39.4% were state-owned enterprises. The sample was divided into two parts, sample A (*N*_A_ = 306) and sample B (*N*_B_ = 306), in order to meet the needs of the subsequent factor analysis. The *t*-test results for the independent samples showed that there were no significant differences between sample A and sample B in terms of gender, age, education, years of working, annual income, nature of company shareholding, w position level and religious beliefs.

### 5.2. Item discrimination test

As in the pre-investigation stage, the study aggregated the scores of all items and ranked them from high to low, naming the first 27% as a high group and the last 27% as a low group. The means of the scores for each item in these two groups were then tested and the results are shown in Table 4. The level of difference between the means of the 32 items in two groups was significant at the 1% level and the standard deviation of the scores for each item was greater than 0.50. In summary, the item differentiation of the scale is satisfactory.

**Table 4:** Analysis of the difference in mean and standard deviation of the items.

Item	Standard deviation	Mean		Sig.	Item	Standard deviation	Mean		Sig.
		Low	High				Low	High	
V1	1.524	1.78	3.28	0.00	V17	1.863	1.93	4.84	0.00
V2	1.304	1.45	3.02	0.00	V18	1.713	1.82	4.60	0.00
V3	1.420	1.41	3.25	0.00	V19	1.855	1.98	4.90	0.00
V4	1.508	1.58	3.61	0.00	V20	1.617	1.55	4.42	0.00
V5	1.685	2.55	4.72	0.00	V21	1.453	1.64	4.18	0.00
V6	1.074	1.16	2.30	0.00	V22	1.371	1.52	3.59	0.00
V7	1.829	2.31	4.72	0.00	V23	1.514	1.62	4.24	0.00
V8	1.648	1.62	4.08	0.00	V24	1.402	1.58	3.78	0.00
V9	1.165	1.15	2.65	0.00	V25	1.588	1.67	4.46	0.00
V10	1.433	1.38	3.59	0.00	V26	1.510	1.73	4.16	0.00
V11	1.452	1.30	3.68	0.00	V27	1.443	1.55	3.79	0.00
V12	1.359	1.31	3.38	0.00	V28	1.593	1.62	4.25	0.00
V13	1.930	1.68	4.43	0.00	V29	1.406	1.38	3.71	0.00
V14	1.877	1.74	4.48	0.00	V30	1.392	1.36	3.68	0.00
V15	1.841	1.85	4.67	0.00	V31	1.448	1.38	3.92	0.00
V16	1.898	1.97	4.88	0.00	V32	1.445	1.37	3.87	0.00

### 5.3. Explanatory factor analyses

Prior to conducting the validation factor analysis, this study examined whether the design of the items met the quality requirements. This study used sample A for the interpretive factor analysis and the results of the KMO, and the results of KMO and Bartlett tests indicated that the sample was suitable for factor analysis (KMO = 0.93, Sig. = 0.00). The research used PCA to extract common factors. The number of factors extracted was fixed at 8. The factor-loading matrix was obtained by the maximum variance method. The cumulative explained variance was 78.84%.

There are two main factors analysis indicators used to test the quality of items, as item commonality and factor loadings. The item commonality reflects the degree of relationship between the original items and the common factors. The greater the item commonality, the higher the degree of explanation of the common factor extracted for each original item. It is generally accepted that the item commonality should not be lower than 0.50 (Pan et al., 2014). In a statistical sense, the factor loadings index is the correlation coefficient between the original items and the common factor, which reflects the relative importance of the items on the common factor. Typically, factor loadings are subjected to a rejection threshold of 0.50 (Pan et al., 2014). At the same time, the cross-factor loading coefficient should not exceed 0.40 to ensure the discrimination validity of the item design (Weng et al., 2018). The results of the indicator tests are shown in Table 5. It can be seen that of all 32 items, item V10 was removed due to its commonality was below 0.5, and items V6 and V18 were removed due to their cross-factor loading were greater than 0.40. The final remaining 29 items were well distributed across all factors.

**Table 5:** The analyzing matrix of item factors.

Item	Commonality	Factors			Item	Commonality	Factors			Item	Commonality	Factors	
		S1	S2	S3			S4	S5	S6			S7	S8
V1	0.62	0.72			V13	0.68	0.74			V25	0.83	0.74	
V2	0.82	0.86			V14	0.77	0.84			V26	0.84	0.75	
V3	0.81	0.84			V15	0.80	0.83			V27	0.86	0.75	
V4	0.72	0.74			V16	0.80	0.80			V28	0.84	0.80	
V5	0.67		0.72		V17	0.78		0.77		V29	0.93		0.82
V6	0.71	Cross factor loadings，removed			V18	0.84	Cross factor loadings，removed			V30	0.91		0.78
V7	0.69		0.77		V19	0.73		0.58		V31	0.93		0.81
V8	0.70		0.68		V20	0.74		0.66		V32	0.93		0.81
V9	0.78		0.79		V21	0.85			0.70				
V10	0.44	Low commonality, removed			V22	0.92			0.78				
V11	0.80			0.68	V23	0.87			0.76				
V12	0.74			0.70	V24	0.91			0.77				
Name of factors		Moral justification	Euphemistic labeling	Advantages comparison			Displacing responsibility	Diffusing responsibility	Misconstruing consequence			Hierarchical order	Blame attribution

Factors S1–S8 are the eight first-order conceptual categories mentioned previously in this paper.

### 5.4. Confirmatory factor analysis

#### 5.4.1. First-order confirmatory factor analysis

The purpose of implementing a validating factor analysis was used to test whether the multidimensional structure of the previous theoretical analysis could be supported by another sample of evidence, thus achieving a structural validation of the rationalization of perceptions of occupational fraud. The research used sample B for the validating factor analysis, with a data sample of 306 individuals, and processed the data using Amos 24.0 software. We first conducted a first-order eight-factor confirmatory factor analysis and found that the residuals of items V22 and V29 were highly correlated with the residuals of multiple items and did not meet the residual independence principle of. Therefore, items V22 and V29 were removed, leaving the remaining number of items at 27. Then, a first-order confirmatory factor analysis was performed again.

Two first-order alternative models were developed, M1: first-order single-factor model, which assumes that the 27 items share the same underlying variable—perceived rationalization of occupational fraud. M2: first-order eight-factor model, based on a theoretical analysis and exploratory factor analysis structure, which assumes the moral justification, euphemism labeling, favorable comparison, transfer responsibility, dilute responsibility, emphasis on results, differentiation and guilty victim as eight first-order factors.

Based on the above model specification, this paper set the first order factors as latent variables for the first order confirmatory factor analysis. According to Jackson et al. (2009), the 9 main categories of indicators reported in previous literature on confirmatory factor analysis are as follows: *χ*^2^, *df*, *χ*^2^/*df*, GFI, AGFI, CFI, TLI, RMSEA, SRMR. Particularly, the smaller the chi-square (*χ*^2^) value, the better the overall fit of the model, and the greater the degree of freedom (df), the more compact the model. The *χ*^2^/ *df* measure is a measure of the degree of fit between the theoretical and observed models, after taking into consideration the complexity of the model. Values less than 5 are considered acceptable and values less than 3 are considered ideal. GFI, AGFI, CFI, and TLI are measures of the similarity between the theoretical model (model covariance matrix) and the observed model (sample covariance matrix). In general, values above 0.80 are acceptable and above 0.90 are ideal. Conversely RMSEA and SRMR are indicators of the difference between the theoretical model (model covariance matrix) and the observed model (sample covariance matrix), and in general, with values below 0.08 being typically acceptable and below 0.05 being ideal.

Based on the above criteria, the results of the M1 and M2 are shown in Table 6. It can be seen that none of the indicators meet the acceptable criteria, except for the first-order single-factor model, which is better than the first-order eight-factor model in terms of simplicity, and the *χ*^2^ value is 3,115.26, much higher than that of the first-order eight-factor model. On the other hand, in the first-order eight-factor model, the *χ*^2^/*df*, CFI, TLI, and SRMR indicators all exceeded ideal values, and GFI, AGFI, and RMSEA also exceeded acceptable values. Meanwhile, as shown in Table 7, the loadings factors of the M2 model items on the corresponding factors exceeded the threshold value of 0.5 and were significant at 0.001 level. Therefore, the M2 model in the first-order model is optimal.

**Table 6:** The main fitting index of the occupational fraud rationalization model.

Model	*χ*^2^	*df*	*χ*^2^/*df*	GFI	AGFI	CFI	TLI	RMSEA	SRMR
Ideal values	smaller = better	greater = leaner	<3	>0.9	>0.9	>0.9	>0.9	<0.05	<0.05
Acceptable values			<5	>0.8	>0.8	>0.8	>0.8	<0.08	<0.08
M1: 1st order single-factor	3,115.26	324	9.62	0.50	0.41	0.50	0.46	0.17	0.12
M2: 1st order eight-factor	711.26	296	2.40	0.86	0.82	0.93	0.91	0.07	0.05
M3: 2nd order three-factor	771.92	313	2.47	0.84	0.81	0.92	0.91	0.07	0.06

**Table 7:** Parameter significance estimates for items in M2.

Factor	Items	Loadings	Parameter sig. estimates			
			Unstd.	S.E.	*t*-value	*p*-value
S1	V1	0.71	1.00			
	V2	0.90	1.13	0.08	14.22	^***^
	V3	0.83	1.10	0.08	13.14	^***^
	V4	0.71	1.05	0.09	11.53	^***^
S2	V5	0.63	1.00			
	V7	0.72	1.23	0.15	8.00	^***^
	V8	0.71	1.08	0.13	8.03	^***^
S3	V9	0.50	1.00			
	V11	0.83	2.75	0.32	8.73	^***^
	V12	0.92	2.62	0.32	8.22	^***^
S4	V13	0.70	1.00			
	V14	0.76	1.04	0.09	12.07	^***^
	V15	0.85	1.15	0.09	12.94	^***^
	V16	0.87	1.25	0.09	13.45	^***^
S5	V17	0.70	1.00			
	V19	0.77	1.05	0.11	10.03	^***^
	V20	0.76	0.91	0.09	10.04	^***^
S6	V21	0.82	1.00			
	V23	0.85	1.06	0.07	15.34	^***^
	V24	0.82	0.97	0.06	15.08	^***^
S7	V25	0.80	1.00			
	V26	0.83	0.94	0.06	16.27	^***^
	V27	0.90	0.95	0.05	17.69	^***^
	V28	0.78	0.95	0.06	14.78	^***^
S8	V30	0.90	1.00			
	V31	0.96	1.08	0.04	29.56	^***^
	V32	0.96	1.09	0.04	29.31	^***^

^***^Represents *p* < 0.001.

#### 5.4.2. Second-order confirmatory factor analysis

Based on the above results, this paper further developed the validation of the second-order structure for the cognitive rationalization of occupational fraud perceptions and therefore proposed model M3. A second-order three-factor model included cognitive reconstruction (A1), responsibility distortion (A2) and value devaluation (A3). The results of the second-order model in Table 8 show that the main fitting indicators *χ*^2^/*df*, CFI and TLI reach ideal values, and GFI, AGFI, RMSEA and SRMR reach acceptable values, which suggested the second-order model is a good fit. However, it is important to note, that when there are more than four first-order factors in a second-order model, the specification of the second-order model will inevitably increase the chi-square (*χ*^2^) value, which will result in a reduction in the overall fit of the model. In order to test whether the second-order model adequately represents the first-order, model Marsh and Hocevar (1985) proposed a test for the target coefficient (Eq. 1). That is, by dividing the chi-square value of the first-order model by the chi-square value of the second-order model, the closer the target coefficient obtained is to 1, the more representative the second-order model is.


(1) $$\mathrm{Target coefficient}=\chi^{2}\left(\mathrm{first order}\right)/\chi^{2}\left(\mathrm{second order}\right)$$

**Table 8:** Reliability and validity test of the first-order latent variables.

	S1	S2	S3	S4	S5	S6	S7	S8
S1	**0.79**							
S2	0.65	**0.69**						
S3	0.49	0.68	**0.77**					
S4	0.36	0.53	0.46	**0.80**				
S5	0.49	0.64	0.64	0.66	**0.74**			
S6	0.42	0.47	0.61	0.44	0.75	**0.83**		
S7	0.53	0.63	0.66	0.43	0.65	0.66	**0.83**	
S8	0.39	0.44	0.51	0.46	0.44	0.45	0.64	**0.94**
AVE	0.63	0.48	0.60	0.64	0.55	0.69	0.69	0.89
CR	0.87	0.73	0.81	0.87	0.79	0.87	0.90	0.96

The bold values on the diagonal are the square root of the latent variable average (AVE), and the rest are the correlation coefficients. Average variation extraction (AVE). Composite reliability (CR).

**Figure 1:**
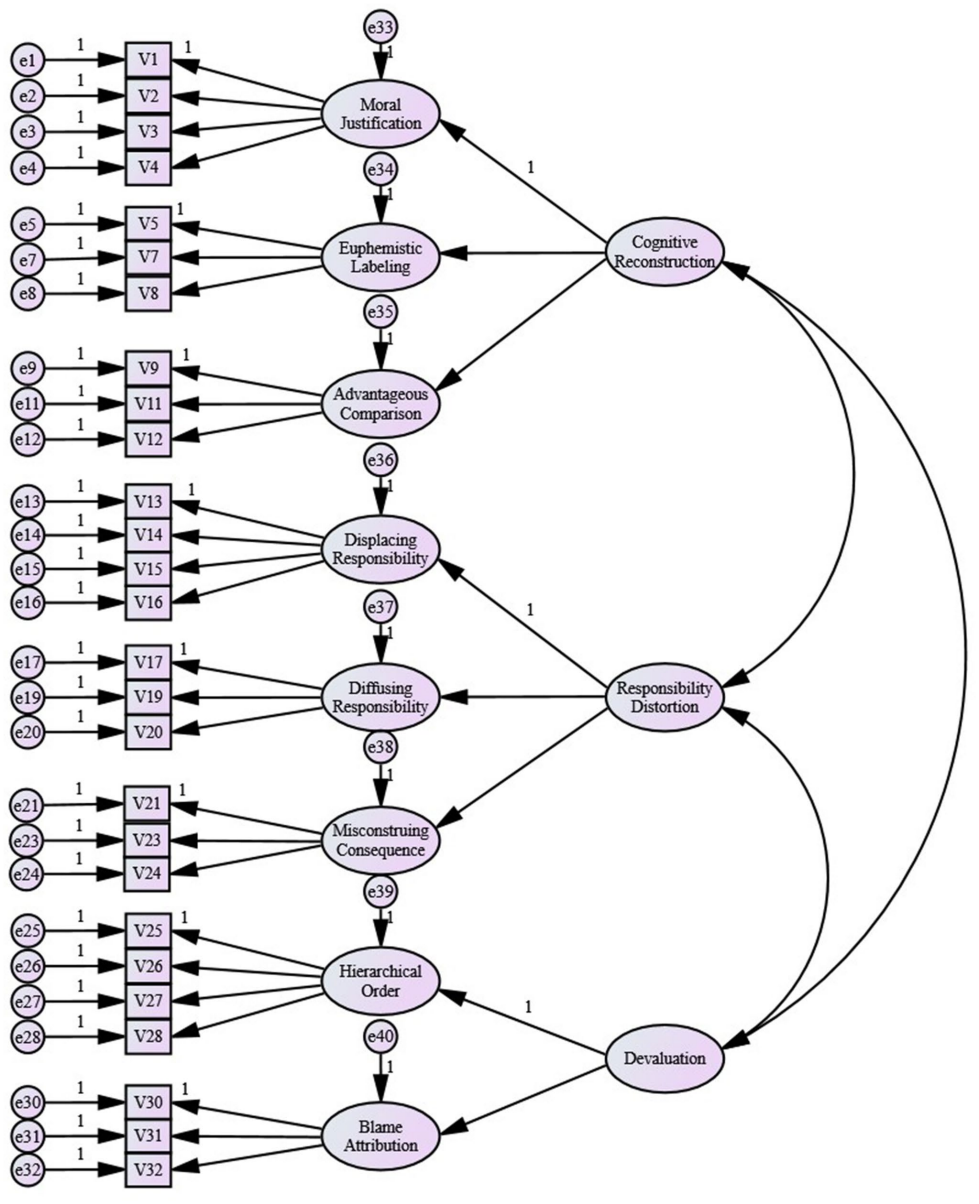
The second-order three-dimensional structure model of occupational fraud rationalization.

Based on the above criteria, the target coefficient for the second-order model in this paper is 0.92 (711.26/771.92). Doll et al. (1994) argued that a target coefficient of 0.74 would provide reasonable evidence for the representativeness of the second-order model. Therefore, the second-order model for rationalizing occupational fraud in this study is sufficiently representative and its structure is demonstrated, as shown in Figure 1.

### 5.5. Reliability and validity test

The assessment of scale reliability is divided into two parts, the overall reliability of the scale and the reliability of the latent variable (Pan et al., 2014). In this case, the overall reliability of the scale is expressed by the Cronbach *α* coefficient, with values greater than 0.70 being an acceptable criterion (Nunnally, 1978). The reliability of latent variables is expressed in terms of composite reliability (CR) value to measure the level of internal consistency between the latent variables and the observed items, preferably greater than 0.60 (Bagozzi and Yi, 1988). After analysis, the overall Cronbach *α* coefficient for the scale was 0.93, indicating that the overall reliability of the scale was high. The reliability tests of the first-and second-order latent variables are shown in Tables 8, 9, respectively, where the CRs of the latent variables of S1 to S8 and A1 to A3 were all greater than 0.60, supporting the reliability of the scale.

**Table 9:** Reliability and validity test of the second order latent variables.

	A1	A2	A3
A1	**0.78**		
A2	0.78	**0.81**	
A3	0.75	0.53	**0.87**
AVE	0.61	0.66	0.75
CR	0.82	0.85	0.85

The bold values on the diagonal are the square root of the latent variable average (AVE), and the rest are the correlation coefficients. Average variation extraction (AVE). Composite reliability (CR).

The assessment of the scale validity mainly includes content validity, validity and discrimination validity. In specific, content validity refers to the ability of the scale items to effectively measure the research problems and is generally judged by qualitative methods. In this study, the scale items were developed through a series of rigorous control measures such as data collection, research interviews, content self-inspection, review & validation and pre-investigation purification. Therefore, the content validity of the scale items was reliable.

The structural validity of a scale is divided into convergent validity and discrimination validity. In specific, the test of convergent validity is determined by average variation extraction (AVE). The higher the AVE value the higher the correlation between the internal measures of the latent variables, as the better the convergent validity of the latent variables. According to Fornell and Larcker’s (1981) criterion, an AVE value greater than 0.50 indicates good convergent validity of the latent variable. In terms of convergent validity, the AVE values of the first-and second-order latent variables were higher than 0.50, except for variable S1 whose AVE value (0.48) was slightly lower than 0.50, indicating good overall convergent validity of the scale. As far as discrimination validity, the AVE square root of the first-and second-order latent variables was no lower than the correlation coefficient between that latent variable and the other variables, except for the AVE square root of variable S5 (0.74), which was slightly lower than the correlation coefficient between S5 and S6 (0.75). This indicates that the overall discrimination validity of the scale is better. In summary, with the exception of the validity of the calibration scale, the scale as a whole passed the validity test.

## 6. Empirical test of criterion validity of occupational fraud rationalization

Calibration validity provides evidence for the validity of a scale construction in terms of the correlation relationships between the scale variables and other variables. Other variables that are empirically related to the variables measured by the scale are referred to as “associated calibrations.” The selection of an associated calibration does not require a causal relationship between it and the variables measured by the scale. It only requires theoretical or empirical evidence that there is a correlation relationship between the calibration or “standard” and the scale variables (Xi, 2016). Considering the implicit nature of occupational fraud rationalization, it is often closely related to the personality traits of the perpetrator. In view of this, and based on the study of previous theoretical and empirical relationships, this paper selects “Machiavellian traits” and “empathy traits” as the associated calibration to test the calibration validity of occupational fraud rationalization.

### 6.1. The selection of associated calibrations

#### 6.1.1. Machiavellian personality and occupational fraud rationalization

Machiavellianism belongs to a group of personality traits that are oriented towards the pursuit of self-interest, manipulative and unscrupulous (Christie and Geis, 1973). In evolutionary psychology, Machiavellianism, Narcissism and Psychopathy are collectively called as the Dark Triad of personality. High Machiavellians have classical traits in behavior and psychology like (1) indifference and lack of empathy, (2) being conspiracy and adopting a calculating and analytical attitude towards other people and situations, (3) being extremely self-focused and having self-interest and self-goals as the sole criteria (4) being manipulative and having a habit of using others as manipulative tools when dealing with interpersonal relationships (Qin and Xu, 2013). In the specific context of occupational fraud, Murphy (2012) used an experimental approach to simulate a reporting environment to examine the feelings and fraudulent decision-making performance of actors with different Machiavellian tendencies after making a false report. The results showed that individuals with high-Machiavellian traits would show lower degrees of guilt after misreporting and a higher frequency and financial amount of fraud than others. In other words, high Machiavellians do not have a strong sense of self-blame for ethical violations in committing fraud, and as a result, their motivation and propensity to implement rationalization to mitigate inner condemnation is reduced. Specifically, the adoption of conspiratorial thinking makes them good at selectively exploiting favorable information in a situation and using it to reconstruct perceptions to achieve motivation to perpetrate occupational fraud. Extremely egocentric personality traits allowed high Machiavellians to habitually shirk responsibility, thereby displacing, distracting and misinterpreting their own responsibilities and making them inclined to view victims of occupational fraud as tools to achieve their own interests, which leads to their devaluation. According to the above analysis, actors with a high degree of Machiavellian traits should have a higher tendency to rationalize occupational fraud perceptions, i.e., there is a positive correlation between an actor’s degree of Machiavellian traits and the rationalization of occupational fraud perceptions.

#### 6.1.2. Empathy traits and occupational fraud rationalization

The term “empathy” has been developed in the field of social psychology for over a century, and the consensus that has emerged is that individuals with high empathy traits are better at understanding the states and feelings of others and creating emotional resonance (An et al., 2018). Furthermore, empathy can be divided into two components (Gladstein, 1983), namely emotional empathy and cognitive empathy. Of these, the former represents the recognition of and response to the emotions of others, while the latter represents the understanding of the causes of the emotional states of others. Accordingly, occupational fraud rationalization arises primarily from ignoring or misrepresenting the emotions, needs and opinions of others (Moore et al., 2012). In this way, individuals with high empathy traits can significantly inhibit rationalization thoughts due to their strong ability to recognize, feel and understand emotions (Chowdhury and Fernando, 2014). When it comes to the specific dimensions of occupational fraud rationalization, an individual’s high cognitive and empathy ability to discriminate between the cognitive reconstruction and responsibility distortion components of occupational fraud rationalization leads to a higher degree of discrimination, thereby inhibiting the operation of above two pathways. An individual’s high affective empathy will strengthen his empathic response to victims of occupational fraud, thereby refusing to rationalize occupational fraud by devaluing them. According to the above analysis, an actor’s high empathy traits will significantly inhibit his acceptance and identification with the occupational fraud rationalization, as there is a negative correlation between the degree of the actor’s empathic trait and the cognitive rationalization of occupational fraud.

### 6.2. Research method

#### 6.2.1. Data

For the third questionnaire in this study, the formal scale for rationalizing occupational fraud, validated by the structure above, was used, excluding those who had already participated in previous surveys. The rejection criteria for the recovered sample were the same as before, and a final valid sample size of 357 was obtained. The structure of the sample was as follows: from the gender perspective, 39.8% were male and 60.2% were female. From the perspective of age, 9% were aged 18 to 25, 27.5% were aged 25 to 30, 36.1% were aged 30 to 40, 17.1% were aged 40 to 50, and 10.4% were aged 50 and above. From the perspective of education level, 15.9% were college and below, 35.0% were undergraduates and 49% were postgraduates. From the perspective of working years, 54.5% were under 10 years, 23% were over 10–20 years and 22.4% were over 20 years. From the perspective of work position level, 63.6% were general employees and 36.4% were managers and senior managers. From the perspective of annual income, 46.5% were below ¥100,000, 33.6% were between ¥100,000 and ¥200,000, 9.8% were between ¥200,000 and ¥300,000 and 10.1% were above ¥300,000. From the perspective of religion, 90.2% were non-religious and 9.8% were religious. From the nature of the shareholding of the companies to which they belong, 63.6% are non-state enterprises and 36.4% are state enterprises.

#### 6.2.2. Measurement tools

The third questionnaire measures basic statistical information, occupational fraud rationalization, Machiavellian personality, empathy traits and social desirability effects of the respondent. The scales were rated on a 7-point Likert scale, except for basic statistical information and social desirability effects.

Machiavellian personality was measured using a scale developed by Dahling et al. (2009). Items from five dimensions of unethical manipulation were used in the measurement process, such as such as “I talk to others for the reason of getting information that is beneficial to me,” etc. The Cronbach *α* coefficient of this scale in this study was 0.76.

Empathy traits were measured using the interpersonal reactivity scale developed by Davis (1980). The empathetic care dimension measures an individual’s capacity for emotional empathy and includes 6 items, such as “I often feel soft-hearted, thoughtful, and caring for those less fortunate than I am,” etc. The perspective dimension measures an individual’s cognitive empathy and includes 5 items such as “Before I make a decision, I try to think about the problem from everyone’s point of view,” etc. The Cronbach *α* coefficient of this scale in this study was 0.811. According to previous studies on occupational fraud, this paper selected demographic variables such as gender, age, education level, years of working, position level, income level, and religion as well as industry-level variables such as equity nature, industry as control variables (Kong and Chen, 2016; Chen et al., 2017; Wang et al., 2018).

The same scales and scoring criteria as in the pre-experiment were used to control for social desirability effects.

#### 6.2.3. Test models

In order to test the relationship between associated calibrations and occupational fraud rationalization, the following model was developed for this study: where Eq. (2) was used to test whether there was a positive relationship between the actor’s Machiavellian traits and occupational fraud rationalization when the social desirable effects and other control variables were controlled for. Eq (3) was used to test whether there was a negative relationship between the actor’s empathy traits and occupational fraud rationalization when the social desirable effects and other control variables were controlled for.


(2) $$Rat_{i}=\alpha_{0}+\alpha_{1}Machi_{i}+\alpha_{2}Desira_{i}+\sum \alpha Control_{i}+\varepsilon_{i}$$

(3) $$Rat_{i}=\beta_{0}+\beta_{1}Emp_{i}+\beta_{2}Desira_{i}+\sum \beta Control_{i}+\varepsilon_{i}$$

In the above models, *Rat* refers to the overall level variable of occupational fraud rationalization, *Desira* represents social desirable effect and *Control* represents the control variables. On the basis of Eqs. (2) and (3), this paper further tested the relationship between Machiavellianism, empathy traits and variables of second-order factor levels (A1 to A3) and first-order factor levels (S1 to S8) of occupational fraud rationalization. The detailed definitions of the variables in the model are given in Table 10.

**Table 10:** Definition of variables.

Variables			Abbreviations	Definition
Dependent variables	Occupational fraud rationalization		*Rat*	Mean of the items
	Second-order three-factor	Cognitive reconstruction	*A1*	
		Responsibility distortion	*A2*	
		Value devaluation	*A3*	
	First order-eight-factor	Moral justification	*S1*	
		Euphemistic labeling	*S2*	
		Favorable comparison	*S3*	
		Transfer responsibility	*S4*	
		Dilute responsibility	*S5*	
		Emphasis on results	*S6*	
		Differentiation	*S7*	
		Guilty victim	*S8*	
Independent variables	Machiavellianism		*Machi*	
	Empathy traits		*Emp*	
Control variables	Gender		*Gender*	Male equals 0, female equals 1
	Age		*Age*	Under 18, 18–25, 25–30, 0–40, 40–50, 50–60, 60 and above takes the value of 1–7 respectively
	Education level		*Educa*	High school and below, junior college, undergraduates, postgraduates takes the value of 1–4 respectively
	Work years		*Work_ s*	Under 1 year, 1–3, 3–6, 6–10, 10–15, 15–20, 20 and above takes the value of 1–7 respectively
	Position rank		*Rank*	Ordinary staff, management, senior management takes the value of 1–3 respectively
	Revenue level		*Rev*	Annual salary below 50,000, 50,000–70,000, 70,000–100,000, 100,000–150,000, 150,000–200,000, 200,000–300,000, 300,000 and above takes the value of 1–7 respectively
	Religious faith		*Faith*	Non-religious faith equal 0, with religious faith equal 1
	Equity nature		*Equity*	Non-state owned equals 0, state-owned equals 1
	Social desirable effects		*Desira*	Mean of the items of social desirable effects scale
	Industry		*Industry*	Set up 18 dummy variables according to the 2017 *National Economic Industry Classification* and data structure of the National Bureau of Statistics

### 6.3. Empirical results

#### 6.3.1. Relevant analysis

Without controlling for various demographic variables, this paper first used the Spearman’s correlation test to provide preliminary evidence for a correlation between Machiavellianism, empathy traits and occupational fraud rationalization. The correlation coefficients between the variables are shown in Table 8. The correlation data in Table 11 shows that there is a positive correlation between Machiavellian personality and occupational fraud rationalization (*r* = 0.45, *p* < 0.01) and a negative correlation between empathy and occupational fraud rationalization (*r* = −0.14, *p* < 0.05). The above results tentatively explain the theoretical expectation between the occupational fraud rationalization and associated calibration was valid and provided preliminary evidence for the criterion validity of the occupational fraud rationalization scale as constructed. Among the control variables, none were significant, except for a negative relationship between gender and occupational fraud rationalization (*r* = −0.11, *p* < 0.1).

**Table 11:** Spearman correlation test between variables.

	*Rat*	*Machi*	*Emp*	*Gender*	*Age*	*Educa*	*Work_ s*	*Rank*	*Rev*	*Faith*	*Equity*
*Rat*	1.00										
*Machi*	0.45^***^	1.00									
*Emp*	−0.14^**^	−0.13^*^	1.00								
*Gender*	−0.11^*^	−0.13^*^	0.01	1.00							
*Age*	−0.04	−0.12^*^	0.02	−0.16^**^	1.00						
*Educa*	0.09	0.16^**^	0.06	0.03	−0.31^***^	1.00					
*Work_ s*	−0.05	−0.13^*^	0.05	−0.19^***^	0.89^***^	−0.37^***^	1.00				
*Rank*	−0.05	−0.01	0.07	−0.27^***^	0.49^***^	−0.09	0.53^***^	1.00			
*Rev*	0.07	0.11^*^	0.10^*^	−0.31^***^	0.27^***^	0.17^***^	0.29^***^	0.50^***^	1.00		
*Faith*	−0.07	−0.03	0.02	0.04	0.13^*^	−0.03	0.16^**^	0.14^**^	0.00	1.00	
*Equity*	0.01	0.03	0.01	−0.07	0.06	0.03	0.03	−0.08	0.06	−0.06	1.00

^***,**,*^Represents significant at 0.001, 0.05, 0.01 level, respectively.

#### 6.3.2. Regression analysis

In order to test the hypotheses, this study conducted a multiple linear regression analysis with occupational fraud rationalization as the explanatory variable and Machiavellianism and empathy as the explanatory variables. The results in Table 12 indicate that there is a significant positive relationship between Machiavellian personality and occupational fraud rationalization that is significant at the 1% level, with or without the inclusion of control variables (uncontrolled: *α* = 0.409, *p* < 0.01; controlled: *α* = 0.408, *p* < 0.01). A significant negative correlation was found between empathy traits and rationalization of occupational fraud, and was significant at the 5% level (uncontrolled: *β* = −0.161, *p* < 0.05; controlled: *β* = −0.156, *p* < 0.05). Therefore, the above theoretical expectations were tested and the validity of the occupational fraud rationalization scale was good.

**Table 12:** Regression results of overall level model of occupational fraud rationalization.

Variable name	Associated calibration: Machiavellianism		Associated calibration: Empathy traits	
	*Rat*	*Rat*	*Rat*	*Rat*
*Machi*	0.409^***^ (9.08)	0.408^***^ (9.54)		
*Emp*			−0.161^**^ (−2.14)	−0.156^**^ (−2.01)
*Gender*		−0.102 (−0.97)		−0.197^*^ (−1.70)
*Age*		−0.026 (−0.32)		−0.013 (−0.13)
*Educa*		0.005 (0.07)		0.080 (1.00)
*Work_ s*		0.040 (0.75)		0.012 (0.19)
*Rank*		−0.179^**^ (−2.01)		−0.179^*^ (−1.65)
*Rev*		0.045 (1.28)		0.067^*^ (1.68)
*Faith*		−0.107 (−0.58)		−0.124 (−0.61)
*Equity*		−0.090 (−0.86)		−0.113 (−0.96)
*Desira*		0.176 (0.56)		0.509 (1.45)
*Industry*		Controlled		Controlled
*_cons*	1.121^***^ (7.29)	1.036^*^ (1.91)	3.396^***^ (8.48)	2.628^***^ (3.92)
*N*	357	357	357	357
*R^2^*	0.209	0.252	0.015	0.086
*Adj R^2^*	0.206	0.190	0.012	0.011

^***,**,*^Represents significant at 1% level, 5% level and 10% level respectively, and the values reported in brackets are the *t*-values for corresponding regression coefficients.

With regard to the control variables, in both sets of regression results, position rank was significantly and negatively associated with rationalization of occupational fraud. The possible reason for this was that the higher the manager’s rank, the greater the number and impact of frauds involved and, as a result, the weaker the acceptance of the occupational fraud rationalization is weakened. Gender has a significant negative correlation with the occupational fraud rationalization. Although it was not significant in the Machiavellian group, the was still negative. The findings showed that female employees had lower levels of occupational fraud rationalization than male employees. Similarly, income in the empathy trait group had a significant positive relationship with occupational fraud rationalization, though it was not significant in the Machiavellian group, it was still positive, which in general indicated that the income level of an individual, the greater his or her propensity to accept occupational fraud rationalization. For the remaining non-significant variables, the possible trends in terms of the sign of the regression coefficients are as follows. The higher the education level and the longer the working years, the higher the propensity to accept occupational fraud rationalization. The older the employees with religious faith and state-owned enterprise group, the lower their propensity to accept occupational fraud rationalization.

#### 6.3.3. Additional analysis

This paper further refined the second-order and first-order factor-level analyses of cognitive rationalization of occupational fraud, i.e., replacing the dependent variable with a second-order or first-order factor level variable and keeping the remaining independent and control variables unchanged. The regression results for the second-order factor level model of occupational fraud rationalization are shown in Table 13. It can be seen that Machiavellianism has significant positive correlations with the cognitive reconstruction, responsibility distortion, and devaluation factors (A1: *α* = 0.400, *p* < 0.01; A2: *α* = 0.436, *p* < 0.01; A3: *α* = 0.394, *p* < 0.01). There was a significant negative correlation between empathy traits and the responsibility distort as well as devaluation factors (A2: *β* = −0.202, *p* < 0.05; A3: *β* = −0.187, *p* < 0.05). Although the correlation between empathy traits and the cognitive reconstruction factor was not significant, it was still negative (A1: *β* = −0.121, *p* > 0.1). Overall, the criterion validity of the occupational fraud rationalization remained valid at the second-order factor level.

**Table 13:** Regression results of the second-order factor level model for occupational fraud rationalization.

Variable name	Associated calibration: Machiavellianism			Associated calibration: empathy traits		
	*A1*	*A2*	*A3*	*A1*	*A2*	*A3*
*Machi*	0.400^***^ (7.95)	0.436^***^ (7.46)	0.394^***^ (7.66)			
*Emp*				−0.121 (−1.62)	−0.202^**^ (−2.02)	−0.187^**^ (−2.44)
Control variables	Controlled	Controlled	Controlled	Controlled	Controlled	Controlled
*_cons*	0.652(1.22)	1.525^**^(2.08)	0.715(1.37)	2.047^***^ (3.00)	3.417^***^ (3.86)	2.448^***^ (3.91)
*N*	357	357	357	357	357	357
*R^2^*	0.273	0.195	0.240	0.113	0.081	0.096
*Adj R^2^*	0.213	0.129	0.178	0.040	0.005	0.022

*** means 1% significant level, ** means 5% significant level, * means 10% significant level.

The regression results of the first-order factor model of occupational fraud rationalization are shown in Tables 14, 15. It can be seen that there was a significant positive correlation between Machiavellianism and the eight factors in the first-order level, except for the dimensions of moral justification (S1) and transfer responsibility (S4). Significant negative correlations existed between the empathy traits and the remaining six factors in the first-order level. It is worth noting that, in addition to the non-significant results, there was a change in the sign of the regression coefficients between empathy traits and moral justification (S1) and transfer responsibility (S4). Possible reasons for this are as follows: as mentioned earlier, the empathy traits essentially involves a greater capacity to understand the states and feelings of others and to develop emotional resonance. However, in the case of moral justification, what the actor is faced with is actually a trade-off between the choice of moral behavior under different definitions. In other words, moral justification is essentially a moral dilemma. Therefore, the actor’s empathy traits have an impact on the moral behavior in the dilemma, so that they cannot play their proper role. In term of transfer responsibility, in the corporate context, the transferring responsibility often comes from coercive instructions from superiors or leaders. Under the coercion of superior authority, it is often difficult for the individual’s self-will to function as an effective restraint or constraint, which may lead to non-significant regression results. Overall, criterion validity was verified at the first-order level of occupational fraud rationalization.

**Table 14:** Regression results of the first-order factor level model for occupational fraud rationalization (*Machi*).

Variable name	Associated calibration: Machiavellianism							
	*S1*	*S2*	*S3*	*S4*	*S5*	*S6*	*S7*	*S8*
*Machi*	0.339^***^ (4.95)	0.515^***^ (7.65)	0.347^***^ (6.38)	0.348^***^ (4.24)	0.513^***^ (7.15)	0.447^***^ (7.69)	0.426^***^ (7.38)	0.361^***^ (6.25)
Control variables	Controlled							
*_cons*	1.213 (1.50)	0.734 (0.98)	0.009 (0.02)	2.213^**^ (2.01)	1.809^**^ (2.06)	0.554 (0.92)	0.652 (1.12)	0.777 (1.30)
*N*	357	357	357	357	357	357	357	357
*R^2^*	0.148	0.263	0.208	0.115	0.173	0.241	0.215	0.190
*Adj R^2^*	0.078	0.202	0.143	0.042	0.105	0.179	0.150	0.124

*** means 1% significant level, ** means 5% significant level, * means 10% significant level.

**Table 15:** Regression results of the first-order factor level model for occupational fraud rationalization (*Emp*).

Variable name	Associated calibration: empathy traits							
	*S1*	*S2*	*S3*	*S4*	*S5*	*S6*	*S7*	*S8*
*Emp*	0.001 (0.01)	−0.182^*^ (−1.67)	−0.184^**^ (−2.42)	0.001 (0.01)	−0.269^**^ (−2.19)	−0.339^***^ (−3.80)	−0.178^**^ (−2.12)	−0.197^**^ (−2.13)
Control variables	Controlled							
*_cons*	1.841^*^ (1.90)	2.665^***^ (2.70)	1.637^**^ (2.53)	2.858^**^ (2.23)	4.201^***^ (4.00)	3.193^***^ (4.30)	2.396^***^ (3.39)	2.500^***^ (3.54)
*N*	357	357	357	357	357	357	357	357
*R^2^*	0.069	0.137	0.102	0.070	0.071	0.135	0.081	0.097
*Adj R^2^*	0.007	0.066	0.028	0.007	0.005	0.064	0.006	0.023

*** means 1% significant level, ** means 5% significant level, * means 10% significant level.

## 7. Research conclusions and discussions

The issue of the structure and measurement of occupational fraud rationalization has been a key barrier to development in theory and practice, and as such, the fundamental question of what occupational fraud rationalization consists of remains confusing at this stage. To this end, this paper completed exploration of the structure and scale development of internal psychological factor, that is occupational fraud rationalization, through multiple steps such as data collection, research interviews, review & check, project purification, structural verification and reliability & validity testing, the main findings are as follows: firstly, from the internal structure, occupational fraud rationalization presented a three-dimensional eight-factor internal structure. The second-order and three dimensions were the cognitive reconstruction dimension (A1), the responsibility distortion dimension (A2), and the value devaluation dimension (A3), where the cognitive reconstruction dimension refers to the cognitive reinterpretation of behavior to make occupational fraud appear acceptable, with first-order aspects including moral justification (S1), euphemistic labeling (S2), and favorable comparison (S3). The responsibility distortion dimension was the avoidance and misrepresentation of one’s responsibility for fraud through diversion, distraction and neglect. Its first-order dimensions included transfer responsibility (S4), dilute responsibility (S5) and emphasis on results (S6). The value devaluation dimension, which devalued the victims of fraud into taking occupational fraud for granted, had first-order aspects involving differentiation (S7) and guilty victim (S8). Furthermore, the main differences between the three second-order dimensions are as follows: the cognitive reconstruction focuses on “the nature of occupational fraud itself,” viewing occupational fraud’s as “not bad” or even “good.” The distortion of responsibility dimension is concerned with “whether the fraudsters themselves be held responsible “. Individuals who engage in responsibility distortion do not deny that occupational fraud is inherently a “bad thing,” but they cover up or erase their responsibilities by deflecting, distracting and ignoring the effects of their actions. The value devaluation dimension focuses on “victim of occupational fraud,” who is taken for granted or deservedly victimized through “discrimination” or “hatred.” Secondly, in terms of construct measurement, this paper constructed a formal scale containing 27 items to measure the occupational fraud rationalization. Of these, moral justification (S1) contains 4 questions, euphemistic labeling (S2) contains 3 questions, favorable comparison (S3) contains 3 questions, transfer responsibility (S4) includes 4 questions, dilute responsibility (S5) consists of 3 questions, emphasis on results (S6) contains 3 questions, differentiation (S7) contains 4 questions and guilty victim (S8) contains 3 questions.

The scale items were developed in strict accordance with theoretical requirements, after pre-investigation and item purification, and were subjected to formal research reliability and validity tests. The final 27 items retained passed the test criteria in terms of item discrimination, overall scale reliability, latent variable reliability, content validity, structure validity (convergent validity, discriminant validity) and calibration validity. It indicated that the overall quality of the occupational fraud rationalization scale was satisfactory. Thirdly, from the perspective of the relationship of variables, this paper empirically tested a number of factors affecting occupational fraud rationalization in terms of personality traits and statistical characteristics. Of these, there was a significant positive correlation between Machiavellian personality and occupational fraud rationalization at the second-order, first-order level. The empathy trait was significantly and negatively related to occupational fraud rationalization at the overall, second-order and first-order levels. It can be seen that the fraudsters with Machiavellian personality belong to a group with a high propensity for occupational fraud rationalization. From a fraud management perspective, these individuals have a weaker capacity for internal moral self-regulation and are more inclined than others to commit occupational fraud when the internal control system of the company fails. In terms of empathy traits, individuals with high empathy have greater internal self-discipline and thus greater willpower to resist temptation. In view of this, companies should pay more attention to the examination of individual empathy in the selection and hiring process, especially in the hiring choices involving core secrets, key authorizations and self-regulatory departments. In terms of control variables, there was a significant negative association between higher ranks, female individuals and occupational fraud rationalization, and a significant positive correlation between income level and rationalization of occupational fraud. The above findings also provide empirical evidence for the relationship between individual factors and occupational fraud rationalization from a demographic perspective. However, there is a limitation of this research, which is about rationalization and neutralization. The fact is rationalization and neutralization are used interchangeably. Rationalization is the person psychological process, but neutralizes is the person internal moral condemnation. For example, the guilty feeling is person’s internal moral condemnation. For the future research, it will emphasize the rationalization and neutralization which affect the occupational fraud more serious.

## Data availability statement

The original contributions presented in the study are included in the article/Supplementary material, further inquiries can be directed to the corresponding author.

## Author contributions

MY: conceptualization, funding acquisition, formal analysis, investigation, methodology, software, visualization, writing—original draft, and writing—review and editing. YC: Conceptualization, Data curation, Investigation, Project administration, Methodology, Software, Supervision, Visualization, Writing – original draft, Writing – review and editing. All authors contributed to the article and approved the submitted version.

## Conflict of interest

The authors declare that the research was conducted in the absence of any commercial or financial relationships that could be construed as a potential conflict of interest.

## Publisher’s note

All claims expressed in this article are solely those of the authors and do not necessarily represent those of their affiliated organizations, or those of the publisher, the editors and the reviewers. Any product that may be evaluated in this article, or claim that may be made by its manufacturer, is not guaranteed or endorsed by the publisher.
