# Supplementary material for: Cognitive rationalization in occupational fraud: structure exploration and scale development
Source: Front Psychol. 2023 Jul 6;14:1112127. doi: 10.3389/fpsyg.2023.1112127 (PMC10356987; doi:10.3389/fpsyg.2023.1112127)
Supplement: Supplementary file 1 [file Table_1.doc]

**Appendix: Items included in the final version of the scale (27 items)**

| V1：When misrepresenting financial statement information can help protect employees from losing their jobs, such behavior should not be condemned. |
| --- |
| V2：If you are committing fraud for the benefit of the company, this is acceptable. |
| V3：If you are committing financial fraud to prevent the company from going bankrupt, this is a form of organizational loyalty. |
| V4：You could be forgiven for embellishing financial statements for the sake of the company's growth |
| V5：In the workplace, accepting gifts is a pattern of favors. |
| V7：Sometimes deliberately withholding true information about your company is an effective strategy for maintaining a competitive edge. |
| V8：In the brutal business battlefield, fabricating information to achieve a company's goals is a business warfare strategy. |
| V9：Temporary misappropriation of public funds for one's own use is trivial compared to financial reporting fraud. |
| V11：Accepting small favors is nothing compared to huge corruption. |
| V12：Taking a secret rebate is nothing compared to a huge bribe. |
| V13：If a superior instigates you to embezzle company's assets, the responsibility for the breach should be borne by the superior. |
| V14：As an enforcer, you should not be held responsible for fraudulent acts in carrying out instructions from your superiors. |
| V15：If it is the superior's decision to whitewash the company's financial statements, you as the enforcer should be exempt from liability. |
| V16：If a superior instructs you to reimburse his for personal expenses, the responsibility for the breach should be borne by the superior. |
| V17：Your personal concerns are meaningless when fraud is prevalent throughout the organizational environment. |
| V19：If your team believes that fraud is the best solution to the problem, then the responsibility for fraud should not fall on you. |
| V20：When your entire business is using your position to take bribes, it is understandable that you are forced to participate. |
| V21：It doesn't matter if you use your position to receive some advantage without hurting anyone. |
| V23：A slight increase in corporate profits is not a big deal as long as the level of misstatement in the financial statements does not affect investors' decisions. |
| V24：If misappropriating assets that are no longer useful to the company, such behavior is acceptable. |
| V25：It is reasonable to exaggerate some information about the company to outsiders as long as it does not harm those close to me. |
| V26：It is understandable to use one's position to assign winning bidders to relatives or friends. |
| V27：There is no need to consider whether financial fraud would harm unrelated persons. |
| V28：It is understandable to deprive some outsiders of their rights to benefit oneself in a highly competitive situation |
| V30：If the company repeatedly delays the agreed salary, it should be reasonable to obtain some compensation by misappropriation of public funds. |
| V31：If the company does not honor your promises, it should not be reprehensible to obtain some compensation through fraud. |
| V32：If the results of your efforts have been ruthlessly exploited by the company, it should not be too much to ask to obtain some compensation through fraud |
